# Prevention of pneumococcal infections: Impact of structured medico-pharmaceutical collaborative management to improve vaccination coverage of at-risk patients (OPTIVACC study): Protocol for a multicenter randomized stepped -wedge study

**DOI:** 10.1016/j.conctc.2025.101462

**Published:** 2025-02-15

**Authors:** Florent Dubois, Emilie Champiot-Bayard, Bogdan Cireașă, Paul Loubet, Jérôme Vallat, Julie Bonnet, Valérie Jacob, Pauline Puyo, Ioana Pînzar, Sarah Théret, Emmanuelle Dubois, Elisabeth Peus, Laurent Giraudon, Clarisse Roux-Marson, Pascale Fabbro-Peray, Géraldine Leguelinel-Blache, Jean-Marie Kinowski

**Affiliations:** a Department of Pharmacy, Nimes University Hospital, Univ Montpellier, Nimes, France; b Desbrest Institute of Epidemiology and Public Health (IDESP), Univ Montpellier, INSERM, Montpellier, France; c Department of Infectious and Tropical Diseases, Nimes University Hospital, Univ Montpellier, Nimes, France; d VBIC, INSERM U1047, University of Montpellier, Nimes, France; e Department of Pharmacy, Montauban Hospital, Montauban, France; f Department of Pharmacy, Saint-Gaudens Hospital, Saint-Gaudens, France; g Department of Pharmacy, Alès Hospital, Alès, France; h Department of Pharmacy, Toulouse Regional University Hospital, Univ Toulouse, Toulouse, France; i Department of Pharmacy, Montpellier Regional University Hospital, Univ Montpellier, Montpellier, France; j Department of Pharmacy, Bagnols-sur-Ceze Hospital, Bagnols-sur-Ceze, France; k Department of Pharmacy, Perpignan Hospital, Perpignan, France; l Department of Pharmacy, Sète Hospital, Sète, France; m Department of Biostatistics, Epidemiology, Public Health and Innovation in Methodology, Nimes University Hospital, Nimes, France; n Department of Law and Health Economics, Univ Montpellier, Montpellier, France

**Keywords:** Infectiology, Pneumococcal infections, Vaccination, Hospital pharmacy

## Abstract

**Background:**

*Streptococcus pneumoniae* causes infections especially in patients with immunodeficiency or specific comorbidities. Most could be avoided through pneumococcal vaccination (PV), but PV coverage is only 20 % in France. Many studies assess methods on vaccination coverage improvement, but none evaluates pharmacist-physician collaboration in hospital on PV coverage of inpatients at-risk of invasive pneumococcal disease (IPD).

**Methods:**

This study is a multicentric stepped-wedged randomized trial involving 12 units in 9 French hospitals (3 university and 6 local) during 4 periods of 90 days each. Three clusters will be made, each composed randomly of clinical and surgical units from one university hospital and clinical and surgical units of 2 local hospitals. For each period, one unit will have to include 16 non-vaccinated inpatients at risk of IPD. Patients in the control phase will receive usual care. During the interventional phase, the pharmacist will inform the physician on PV necessity, who will report recommendation and prescribe it at discharge. The pharmacist will perform a consultation and send a discharge letter to the patient's community pharmacist. The primary outcome will assess the impact of intervention on PV coverage after 6 months. Secondary outcomes will evaluate vaccines dispensing, uncompleted protocol rate and intervention process. A subgroup analysis between university and local hospitals and clinical and surgical units, respectively will be made.

**Discussion:**

This study will assess the impact of medico-pharmaceutical collaboration in hospital on PV coverage in inpatients at risk of IPD. Hospitalization could be a way to promote vaccination and enhance healthcare system performance.

**Trial registration:**

Clinicaltrials.gov, NCT05060146. Registered on September 16th, 2021.

## 1 Background

*Streptococcus pneumoniae* (pneumococcus) is a cocci gram-positive bacterium which is responsible for invasive pneumococcal diseases (IPD) like meningitis and bacteremia or more common infections like otitis and community acquired pneumonia. In France, in 2020, pneumococcus was the first cause of bacteremia (5.2 cases/100,000 inhabitants) and meningitis (0.7 cases/100,000 inhabitants) [1]. It is also the main cause of bacterial pneumonia [2]. Children under 5 years old and elders are more likely to develop pneumococcal infections [1,3,4], but other populations are considered at risk, like adult patients with immunodeficiency or patients with some specific comorbidities (cardiac, renal, respiratory failure, diabetes, etc.) [5].

Pneumococcal infections are an economic burden for society. In Europe, all documented cases of pneumonia generate costs around €10 billion per year [6]. In France, the average hospitalization fee for a community acquired pneumococcal pneumonia is €7,293, the follow-up fee being €1241 [7]. Furthermore, the World Health Organization (WHO) included pneumococcus on a priority pathogens list for research and development of new antibiotics, because of the antibiotic resistance of the pathogen, especially against penicillin [8,9]. Pneumococcal pneumonia is also considered the evitable disease with the highest mortality rate [10], explaining that vaccination could be a major health issue.

In France, 2 vaccines against pneumococcus are available: 23-valent pneumococcal polysaccharidic vaccine (PPSV23) and a 13-valent pneumococcal conjugate vaccine (c) [11,12]. In 2017, the High Council for Public Health (HCPH) recommended a sequential vaccination with PCV13 followed by a shot of PPSV23 8 weeks later for all patients older than 5, considered at risk of pneumococcal infections [13,14]. For patients having received just one dose of VPP23, it is recommended to do a dose of PCV13 after 1 year.

Even if the prevention by vaccination seems to be a good solution in view of the economic cost of pneumococcal infections and the emergence of antibiotic resistance, the vaccine coverage is only about 20 % among the target population according to HCPH [14]. A study, conducted in France between 2014 and 2018 presents a rate of only 4.5 % of vaccinated at-risk patients and a decline of vaccination coverage in this period, while patients with chronic pathologies and healthcare visits increase [15]. Some of the main contributing factors include: lack of vaccination campaigns, difficulty in finding patient vaccination history, confusion about prescription and administration of the vaccine between healthcare professionals, lack of knowledge about vaccines among patients and recommendations from physicians, respectively [16,17].

Studies in Canada or the United states show that the pharmacist could promote the PV, both in pharmacies and hospitals for all vaccines. However, none of them were conducted in France, many of the studies are not randomized controlled studies and did not analyze the collaboration between pharmacist and physician [18,19].

The primary outcome of our study is to evaluate the impact of medico-pharmaceutic collaboration in hospital on the PV coverage for at-risk patients. The secondary outcomes are to investigate the rate of incomplete vaccination protocols, vaccines dispensation, multidisciplinary collaboration, as well as a subgroup analysis between different types of units (clinical vs surgical) and healthcare facilities (hospital vs university hospital). Clinical pharmacy practices and the degree of collaboration between caregivers may vary according to the type of service and facility. We want to analyze the collaborative management in different contexts in order to assess these potential differences.

## 2 Methods

### 2.1 Design

**Fig. 1:**
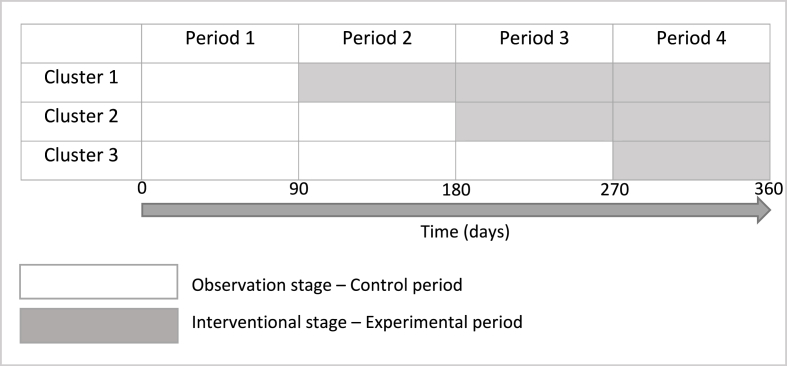
Study design.

This study will be a multicentric prospective stepped wedge controlled randomized trial (SW-CRT) with 3 clusters and 4 periods. This design was chosen because it will not be possible to do an individual randomization of patients. Indeed, there would be a high risk of contamination bias as soon as the interventional strategy will take place in the unit with the medico-pharmaceutical collaboration: it will change the habits for all patients, including control patients. The cluster cross over will be in one direction only. A sequential permutation of each cluster from control to experimental period, determined by randomization, will be made after a set time of 90 days. All the clusters will start in the control phase and end up in the interventional phase. The study design is outlined in Fig. 1.

### 2.2 Settings/population

3 university hospitals and 6 local hospitals with clinical pharmacy activities in the region of Occitanie, France, have agreed to participate in this study. The 3 clusters will be made up of randomly selected hospitals and units. Each cluster comprises 4 units: 2 units (clinical and surgical) from the same university hospital and a clinical and surgical unit from different local hospitals. Hospital centers, units and number of patients to be included are described in Table 1. For this study, each unit will recruit 8 patients per period, so 64 patients during the period of inclusion. Therefore, a hospital will include 64 patients and a university hospital 128, totaling 768 patients for the entire study.

**Table 1:** Hospital centers, units and number of patients to include.

Hospital center	Type of establishment	Unit	Unit type	Total number of patients to include
Nîmes	University hospital	General medicine	Clinical	64
		Digestive surgery	Surgical	64
Montpellier	University hospital	Internal medicine	Clinical	64
		Digestive surgery	Surgical	64
Toulouse	University hospital	Internal medicine	Clinical	64
		Vascular surgery	Surgical	64
Bagnols-sur-Cèze	Hospital	Gastroenterology	Clinical	64
Montauban	Hospital	Pneumology	Clinical	64
Saint-Gaudens	Hospital	General medicine	Clinical	64
Alès	Hospital	Surgery	Surgical	64
Perpignan	Hospital	Surgery	Surgical	64
Sète	Hospital	Surgery	Surgical	64

To qualify, patients must be over 18 years old and covered by the French National Health Insurance (NHI). They must be hospitalized in one of the units participating in this study and also need to be at risk of pneumococcal diseases and eligible to the pneumococcal vaccination according to the French high council of public health recommendations [14]. At admission, patients will receive medication reconciliation from the clinical pharmacist of the department. Patients requiring only a vaccine booster will not be included in the study. The vaccination status of the patient will be confirmed at least by 3 sources as recommended by French society of clinical pharmacy and high authority of health: patient or caregiver declaration, usual drugstore, patient's physician or medical records [20,21]. As last criteria, the patient will go back home at the end of hospitalization. The patient's consent will be obtained and recorded in the medical file, followed by regular monitoring visits carried out by a clinical research associate.

### 2.3 Outcomes

The primary outcome is to evaluate the rates of patients who received a complete protocol of pneumococcal vaccination six months after hospitalization. The coordinating pharmacist of the study will collect this outcome by calling the GP or the home care nurse. The national health data system (SNDS) of the NHI, which lists all medications delivered to patients [22], will also be checked to verify the dispensation of vaccines by community pharmacy. A protocol will be considered complete only if the two vaccines (PCV13 and PPSV23) were administrated.

One of the secondary outcomes will assess prescriptions of pneumococcal vaccination protocol and traceability of the indication of vaccination in the discharge letter. The clinical pharmacist will collect this information in the unit at discharge. Later on, the rates of incomplete vaccination protocol 6 months after the hospitalization will be evaluated. The rate of incomplete protocols will be calculated by dividing the number of patients with incomplete vaccination protocols to the total number of patients, transformed in percents. An incomplete protocol is determined by dispensation and administration of PCV13, not followed by administration of a second injection of PPSV23. As for the primary outcome, the SNDS will be checked and the healthcare staff administrating will be contacted to confirm the administration of the first vaccine but not the second one. The SNDS will also be consulted to analyze the primary dispensation of vaccines by community pharmacy 6 months after hospitalization. The matching between the SNDS data and declarations of community pharmacies about dispensations of vaccines will be also evaluated. The declarations of community pharmacies will be collected after a call by the pharmacist coordinating this study. Finally, a subgroup analysis will be done to compare the difference between clinical and surgical units and university hospitals and hospitals.

### 2.4 Intervention

During the control period, the patient management will be as usual: the pharmacist in the unit will do a medication reconciliation and check his pneumococcal vaccination status. If the patient is eligible for vaccination based on HCPH criteria [14] but not vaccinated according to three sources [20,21], the pharmacist will note it in the patient's medical record. During the month after discharge, the pharmacist of the unit will check pneumococcal vaccines prescription and notification of vaccination indication in the discharge letter by the department physician. Six months after the end of hospitalization, the coordinating pharmacist of the study will call the patient's usual community pharmacy to check vaccine dispensation. He will also call the patient's general practitioner and/or home care nurse to check the vaccine administration. Finally, the SNDS will be analyzed to check all vaccine dispensation.

**Fig. 2:**
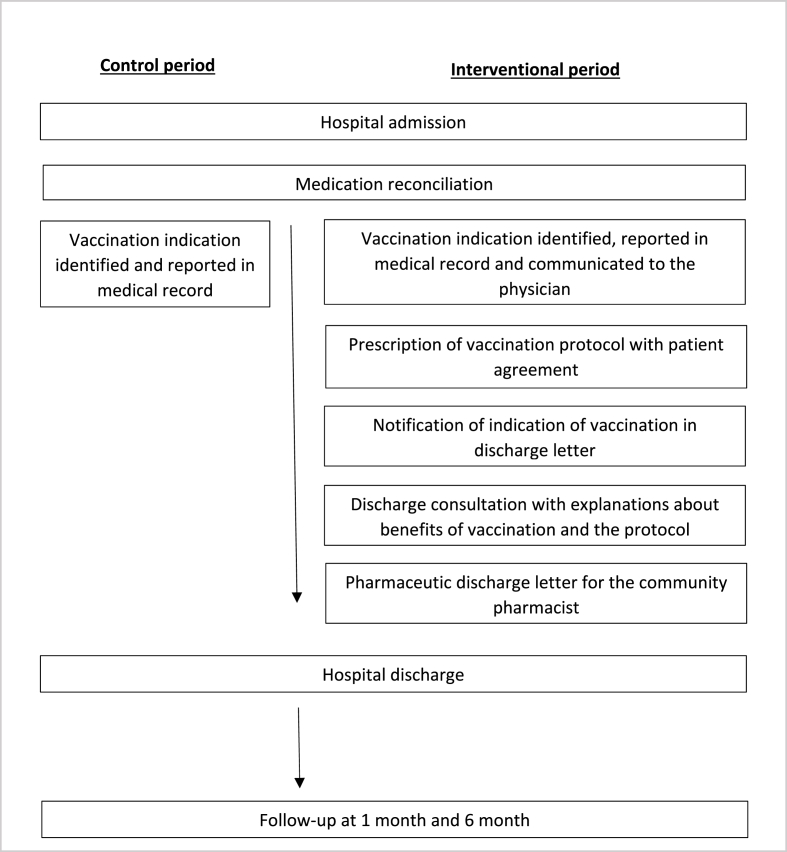
OPTIVACC study - flow of intervention.

Concerning the experimental stage, the pharmacist of the unit will also do medication reconciliation, check the vaccination status of patient and report it in the medical record. If it is negative, the pharmacist will communicate the vaccination benefit to the physician the vaccination benefit. Then the physician will suggest the pneumococcal vaccination and prescribe the protocol with patient agreement. He will note vaccination indication in the discharge letter for the GP. Before the patient returning home, the pharmacist will have a pharmaceutic discharge conversation with the patient and explain the benefits of vaccination and the protocol (dispensation and administration). The pharmacist will also send a pharmaceutic discharge letter to the patient's usual community pharmacy about pneumococcal vaccination. The follow-up procedure and the data collected will be the same for the two phases. For ethical reasons, the coordinating pharmacist will inform the GP of the recommendation to vaccinate all unvaccinated patients of the study at the end of their follow-up. The OPTIVACC study's flow of intervention is outlined in Fig. 2.

### 2.5 Blinding

This study is single-blind. Indeed, the patient will not know if he was included in control period or interventional period. However, due to intervention type and the study design, pharmacists and physicians of the participating units will know if the patient is included in observational or experimental stage because they must do or not the intervention.

### 2.6 Sample size calculation

This study was designed according to a validated methodology of SW-CRT [23]. The Canadian meta-analysis from Taitel et al. suggests that the vaccine coverage risk ratio is 2,96 for intervention [19], but for this study, a less optimistic hypothesis was made.

Considering an intraclass correlation coefficient of 0.05, a power of 95 % and a two-sided alpha level of 5 %, a total of 684 patients must be included to highlight an absolute difference of 20 % between the control group (20 %) and the experimental group (40 %). Assuming 10 % of unusable data, the patient number were increased to 768, so 8 patients per unit and period.

### 2.7 Data management

During this study, RedCap (REDCap 13.4.13 - © 2023 Vanderbilt University, Nashville, Tennessee, USA), a software allowing data collection, will be used. The software will allow access to the database to participants, according their role in the study. Furthermore, it saves any data change. Only authorized people will be able to get the access to electronic case report form (e-CRF), with a login and a password.

The software is hosted by a server of Nîmes University Hospital. All collected data will be saved every day on a secure network. Creation and settings of e-CRF, management during the study and data extraction will be done by the Department of Biostatistics, Epidemiology, Clinical Research and Health Economics.

### 2.8 Statistical analysis

First, descriptive statistics of the population will be done. The quantitative variables will be reported as means and standard deviation or medians and quartiles, while qualitative variables will be expressed as headcounts and proportions.

Concerning the primary outcome, the variable to explain is a complete vaccination protocol. The vaccination rate after 6 months since discharge, between control and interventional, will be analyzed by mixed logistic regression model.

We consider a potential sensitivity analysis performed by age (≥65 years vs ≤ 65 years) and comorbidities (immunocompromised patients vs non-immunocompromised) by sequentially adjusting our statistical model for the potential confounders.

For the secondary outcomes, the matching between the SNDS data and declarations of drugstores about dispensations of vaccines will be access by calculating the kappa coefficient. The other outcomes with binary variable will be parsed by mixed logistic regression model. All statistical analysis above will be stratified by type of units (medicine or surgery) and type of health facility (university hospital or hospital).

Included patients headcount will be described and summarized in a flow chart.

The statistical analysis will be performed by the Department of Biostatistics, Epidemiology, Clinical Research and Health Economics of Nîmes University Hospital by using SAS Enterprise Guide V7.1 (SAS Institute, Cary, NC, USA) or R 3.5.1 (R Development Core Team (200918). R Foundation for Statistical Computing, Vienna, Austria) or a later version.

### 2.9 Dissemination

The scientific committee will be responsible of the publications reporting the results of the study.

## 3 Discussion

This trial will assess the impact of the collaboration between pharmacist and physician on increasing the pneumococcal vaccination of at-risk patients when they return home after hospitalization.

The IP-VAC trial [24], a single center study made by the Nimes University Hospital preliminary to OPTIVACC, showed an increase of vaccines dispensation by community pharmacy. By collecting data on vaccine administration from GPs or home nurses, OPTIVACC study wants to demonstrate an impact on pneumococcal vaccination coverage. To harmonize the data collection, phone calls will be centralized and performed by one officer. In addition, consultation of the NHI database at the end of the study should allow an exhaustive collection of all the vaccine doses administered to the patients.

In addition, consultation of the NHI database at the end of the study should allow an exhaustive collection of vaccines administered to the patients. Intervention will be evaluated in two types of care units (clinical and surgical) and two types of establishments (hospitals and university hospitals). The aim of these comparisons is to define in which context the intervention will be most relevant, in order to target eligible care units.

Patient recruitment will be a critical point of this study, even though there are many indications for pneumococcal vaccination. Firstly, it is difficult to check patients' vaccination status. According to a study carried out as part of the “INNO'vaccins programme”, one patient in two does not know whether he/she is up to date with his/her vaccinations [25]. For this reason, medication reconciliation, a process carried out by the clinical pharmacist to list all patient's medication on hospital admission, is the first step before including a patient in the study.

Although all of the participating centers carry out clinical pharmacy activities, most of them have never conducted clinical research projects. In this sense, initial training of the centers' personal and coordination of the project are two key points for the success of the study, particularly in terms of patients’ recruitment and follow-up.

Patient follow-up will run for a period of six months after discharge to allow the patient to complete the vaccination protocol, which takes at least 8 weeks.

The expectation for this study is to highlight medico-pharmaceutical collaboration in hospitals as a way to improve vaccination coverage. It would optimize drugs management by detecting patients with no history of pneumococcal vaccination, promote awareness about it and encourage an outpatient vaccination process. Furthermore, it would also avoid many IPD and enhance healthcare system performance.

## CRediT authorship contribution statement

**Florent Dubois:** Writing – original draft, Supervision, Methodology, Funding acquisition, Conceptualization. **Emilie Champiot-Bayard:** Writing – original draft, Investigation. **Bogdan Cireașă:** Writing – review & editing. **Paul Loubet:** Methodology, Conceptualization. **Jérôme Vallat:** Investigation. **Julie Bonnet:** Investigation. **Valérie Jacob:** Investigation. **Pauline Puyo:** Investigation. **Ioana Pînzar:** Investigation. **Sarah Théret:** Investigation. **Emmanuelle Dubois:** Investigation. **Elisabeth Peus:** Investigation. **Laurent Giraudon:** Investigation. **Clarisse Roux-Marson:** Project administration. **Pascale Fabbro-Peray:** Validation, Methodology, Formal analysis, Data curation. **Géraldine Leguelinel-Blache:** Writing – review & editing, Supervision, Conceptualization. **Jean-Marie Kinowski:** Supervision, Project administration, Conceptualization.

## Ethics approval and consent to participate

This study will be performed in accordance with the Declaration of Helsinki and has been approved by the committee for the protection of persons (CPP EST IV; reference no. 20/82/SI 20.10.15.61711) and by the French National Agency for the Safety of Medicines (ANSM; reference no. 2020-A01581-38). A letter of non-opposition will be given to all patients recruited.

## Availability of data and materials

All datasets generated from the study are available from the corresponding author.

## Trial status

646 patients were included between 18, September 2022 and 13, September 2023. Follow-up ended on 14, March 2024.

## Funding

This trial is supported by the 2019 GIRCI SOHO PHRC-I call for tenders (Study ID: PHRC-I/2019/FD-01). The study protocol has undergone peer-review by the funding body. The funders have no role on this study in protocol drafting, data collection, data analysis and interpretation.

## Declaration of competing interest

The authors declare that they have no known competing financial interests or personal relationships that could have appeared to influence the work reported in this paper.

## Data Availability

Data will be made available on request.

## Abbreviations

BESPIM: Department of Biostatistics, Epidemiology, Public Health and Innovation in Methodology; COPD: Chronic obstructive pulmonary disease; GP: General Practitioner; PV: pneumococcal vaccine; IPD: invasive pneumococcal disease; HCPH: High Council for Public Health; PCV13: 13 valent pneumococcal conjugate vaccine; PPSV23: 23 valent polysaccharidic vaccine; NHI: National Health Insurance; SNDS: National Health Data System.
