# Supplementary material for: Prevention of pneumococcal infections: Impact of structured medico-pharmaceutical collaborative management to improve vaccination coverage of at-risk patients (OPTIVACC study): Protocol for a multicenter randomized stepped -wedge study
Source: Contemp Clin Trials Commun. 2025 Feb 15;44:101462. doi: 10.1016/j.conctc.2025.101462 (PMC11891599; doi:10.1016/j.conctc.2025.101462)
Supplement: Multimedia component 1 [file mmc1.docx]

#
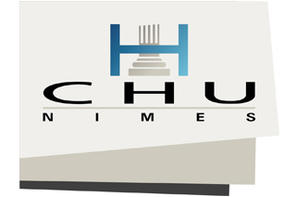
 INFORMATION NOTE FOR PATIENTS

**Study sponsor :**

Nîmes University Hospital

Place du Professeur Debré

30029 Nîmes Cedex 09

The “Optivacc” study

**Prevention of pneumococcal infections: impact of structured medical-pharmaceutical collaborative management to improve vaccination coverage of patients at risk. Multicenter randomized cluster study with sequential permutation (stepped-wedge)**

Version 4.0 of 11/04/2023

ID-RCB number: 2020-A01581-38

Dear Sir/Madam

Your hospital pharmacist has invited you to take part in a research project as part of a public interest mission sponsored by Nîmes University Hospital. Before making a decision, it is important for you to read these pages carefully, as they will provide you with all the information you need on the various aspects of this research. Please do not hesitate to ask your hospital pharmacist should you have any questions.

Your participation is entirely voluntary. If you do not wish to take part in this research, you will continue to benefit from the best possible medical care, in accordance with the current knowledge. Your oral, express, free and informed consent will be proof of your final agreement to take part in the study. Your consent to participate in this research will be recorded in your patient file.

**Presentation OF THE RESEARCH**

In France, pneumococcus is the leading cause of bacterial pneumopathies and meningitis. The frequency and severity of these bacterial infections increase in subjects at risk (for example patients with chronic illnesses, immunosuppressed patients or those undergoing immunosuppressive therapy such as biotherapy or chemotherapy). Pneumococcal vaccination coverage is now available. It takes place in two stages: a first vaccine, the "13-valent", followed by a second vaccine, the "23-valent", at least 8 weeks later. The Haut Conseil de la Santé Publique recommends that at-risk patients undergo pneumococcal vaccination to prevent pneumococcal infections.

The aim of our study is to improve pneumococcal vaccination coverage in these patients by offering them structured collaborative medical-pharmaceutical management.

**RESEARCH PROCESS**

The study we are suggesting you part in takes place in 9 different centers. A total of 768 patients will be included.

During your hospitalization, a hospital clinical pharmacist will come and record the medicinal treatments you are taking (he or she will carry out what we call a " medication conciliation ") according to the usual procedure, and will ensure, for the purposes of the study, that you have not already been vaccinated against pneumococcal disease.

The study is divided into two phases:

- The "Observational" phase: Patients included during this period will receive their usual care.
- The "Interventional" phase: Patients included during this period will benefit, in addition to their usual care, from structured medico-pharmaceutical collaborative care. The aim of this collaborative approach is to promote the need for pneumococcal vaccination before patients are discharged from hospital.

The collaborative approach will take place prior to hospital discharge, as described

below:

- - The physician will provide the patient with a discharge prescription for vaccination.
  - A pharmacist will provide the patient with information about the pneumococcal vaccine, and explain the new treatments.

For all patients, during the 6 months following inclusion, the study investigator will ensure that the two pneumococcal vaccines (13 valent & 23 valent) have been dispensed correctly by your pharmacy, and that they have been administered correctly by your GP or home nurse (if applicable).

Once you have been discharged from hospital, you will no longer be contacted.

**RESEARCH-SPECIFIC PROCEDURES NOT INCLUDED IN STANDARD CARE**

The only intervention that is not part of usual care is structured medico-pharmaceutical collaborative care, which will be reserved exclusively for patients included in the interventional phase of the study.

**reSeArch Population**

The study population corresponds to adult patients admitted for full hospitalization in a surgical or medical ward, who have to undergo medication reconciliation, whose pneumococcal vaccination status is negative and whose risk of pneumococcal infection is high.

**CONSTRAINTS & OBLIGATIONS**

- You must be a member or beneficiary of a health insurance scheme.

- You certify that you are not participating in any ongoing Category 1 research, and that you have not participated in any Category 1 studies in the previous 3 months.

- You certify that you are not up-to-date with your pneumococcal vaccination.

**EXPECTED BENEFIT(S)**

For patients benefiting from collaborative medical-pharmaceutical management, the support offered as part of this study should make it possible to improve their anti-pneumococcal coverage and consequently avoid potentially serious infections.

In general, the medico-pharmaceutical collaboration will also help optimize drug management by improving detection of at-risk patients who have not been previously vaccinated. Better continuity of care can then be ensured in the patient pathway by promoting the smooth running of the outpatient vaccination process.

**FORESEEABLE RISK(S)**

There are no risks involved in participating in this study.

**COMPENSATION**

- No compensation will be paid for this study.
- Interventions specifically added to the usual care for the specific needs of the research will be fully covered. Participation in the research will not incur any additional costs over and above those you would incur for your usual care.

**WHAT ARE YOUR RIGHTS ?**

Your hospital pharmacist must provide you with all the necessary explanations concerning this research. If you wish to withdraw from the study at any time, for whatever reason, you will continue to benefit from medical monitoring, and this will in no way affect your future surveillance.

As part of the research, your personal data will be processed electronically to enable the results of the research to be analyzed regarding the objectives presented to you. The data controller is Nîmes University Hospital. The study doctor and other study personnel will collect information about you, including your health data, concerning your participation in the study. This information, known as "Personal Information", is recorded on forms provided by the sponsor. Only information that is strictly necessary for the treatment and purpose of the research will be collected, and this data will be kept for up to two years after the last publication of the research results or, if no publication has been made, until the final research report is signed. They will then be archived on paper or electronically for a period of 15 years from the end of the study, in accordance with current regulations. To ensure the confidentiality of your personal information, neither your name nor any other information that could directly identify you will be entered on the case report form or in any other file or sample that the study physician provides to the sponsor or the sponsor's authorized representatives. You will be identified only by a code and your initials. The code is used so that the study pharmacist can identify you if necessary.

In accordance with the provisions of the French Data Protection Act (Act no. 78-17 of January 6, 1978 on Data Processing, Data Files and Individual Liberties, as amended by Act no. 2018-493 of June 20, 2018 on the protection of personal data) and the General Data Protection Regulation (EU Regulation 2016/679), you have the following rights with regard to the data we collect as part of this study:

• the right to request information on the processing of your data.

• the right to request rectification of your data if it is inaccurate or incomplete. While we examine your request, you have the right to restrict the processing of your data,

• the right to withdraw your consent or to object to the processing of your personal data, at any time, without having to justify your decision. No further data will be collected after you have withdrawn your consent.

• If you withdraw your consent or object to the processing of your data, you may request the deletion of your data already collected if there is no other legal requirement for their use. Please note, however, that your data that will have already been processed with your initial consent will be retained so as not to make it impossible or compromise the achievement of the research objectives (Articles 17.3.c & 17.3.d. of the General Data Protection Regulation).

You may exercise these rights by writing to the study's hospital pharmacist. The sponsor will respond to your requests as far as possible in accordance with its other legal and regulatory obligations and where required by law.

You also have the right to object to the transmission of data covered by professional secrecy that may be used and processed as part of this research. You also have the right to access all your medical data, either directly or through the doctor or pharmacist of your choice, in accordance with article L1111-7 of the French Public Health Code.

These rights may be exercised with the pharmacist who is following you as part of the research and who is aware of your identity.

The competent authorities and the sponsor or its authorized representatives may also require access to your medical records and study file, in order to verify the data collected in the course of the study.

Your coded personal information may be used for other scientific research on pneumococcal vaccination or vaccination policy, always in compliance with applicable laws and regulations. In this context, research may be carried out by the promoter alone, or with one or more public or private institutions (including outside the European Union), or only by the latter. You may object to this re-use at any time by contacting the doctor who is treating you as part of this research, or the Data Protection Officer.

Should you have any further questions about the collection and use of your personal information, or about your rights in relation to this information, please contact the CHU de Nîmes Data Protection Officer (dpd@chu-nimes.fr) or the study pharmacist. The postal address of the DPO is as follows: DPD, CHU de Nîmes, place du Pr. Debré 30029 Nîmes.

If, despite the measures put in place by the promoter, you feel that your rights have not been respected, you may file a complaint with the competent data protection supervisory authority in your country of residence (the CNIL for France) by writing to the following address: CNIL, TSA 80715, 3 Place de Fontenoy, 75334 Paris or directly on the CNIL website at [www.cnil.fr](http://www.cnil.fr)

In accordance with law n°2012-300 of March 5th, 2012 on research involving human persons:

- this research has obtained a favorable opinion from the Comité de Protection des Personnes EST IV and has been declared to the Agence Nationale de Sécurité du Médicament et des produits de santé (ANSM), an authorization from the CNIL will be requested and the NIR will be collected to query SNIRAM data.

- Nîmes University Hospital has taken out civil liability insurance with HDI GLOBAL SE (n° 0101242214029) (Tour Opus 12, La Défense 9, 77 Esplanade du Général de Gaulle - 92 914 Paris La Défense Cedex),

- people who have suffered prejudice as a result of participating in research can assert their rights before the regional commissions for conciliation and compensation of medical accidents,

- once the research is completed, you will be personally informed of the overall results by your pharmacist as soon as they are available, if you so wish.

After reading this information note, please do not hesitate to ask the hospital pharmacist any questions you may have.

*Thank you for your attention.*

**Should you have any questions, please do not hesitate to ask the study coordinating pharmacist.**

| **Name and contact details of the coordinating investigator** |
| --- |
| Dr Florent DUBOIS  Pharmacy department  CHU de Nîmes  Tel : 04.66.68.31.04 |

**Please be assured that your participation is extremely valuable to us. Thank you in advance for your support of medical research.**

#
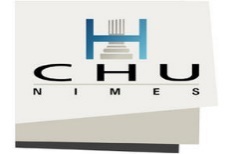
 INFORMATION NOTE FOR THE TUTOR/CURATOR

**Study sponsor :**

Nîmes University Hospital

Place du Professeur Debré

30029 Nîmes Cedex 09

The “Optivacc” study

**Prevention of pneumococcal infections: impact of structured medical-pharmaceutical collaborative management to improve vaccination coverage of patients at risk. Multicenter randomized cluster study with sequential permutation (stepped-wedge)**

Version 4.0 of 11/04/2023

ID-RCB number: 2020-A01581-38

Dear Sir/Madam

We are offering the patient the chance to take part in a research project as part of a public interest mission sponsored by Nîmes University Hospital. Before making a decision, it is important for you to read these pages carefully, as they will provide you with all the information you need on the various aspects of this research. Please do not hesitate to ask your hospital pharmacist should you have any questions.

Your participation is entirely voluntary. If you do not wish the patient to take part in this research, he/she will continue to benefit from the best possible medical care, in accordance with the current knowledge. Your oral, express, free and informed consent will be proof of your final agreement for the patient to take part in the study. Your consent for the patient to participate in this research will be recorded in the patient’s file.

**Presentation OF THE RESEARCH**

In France, pneumococcus is the leading cause of bacterial pneumopathies and meningitis. The frequency and severity of these bacterial infections increase in subjects at risk (for example patients with chronic illnesses, immunosuppressed patients or those undergoing immunosuppressive therapy such as biotherapy or chemotherapy). Pneumococcal vaccination coverage is now available. It takes place in two stages: a first vaccine, the "13-valent", followed by a second vaccine, the "23-valent", at least 8 weeks later. The Haut Conseil de la Santé Publique recommends that at-risk patients undergo pneumococcal vaccination to prevent pneumococcal infections.

The aim of our study is to improve pneumococcal vaccination coverage in these patients by offering them structured collaborative medical-pharmaceutical management.

**RESEARCH PROCESS**

The study we are suggesting that the patient take part in is held in 9 different centers. A total of 768 patients will be included.

During the patient’s hospitalization, a hospital clinical pharmacist will come and record the medicinal treatments he/she is taking (he or she will carry out what we call a " medication conciliation ") according to the usual procedure, and will ensure, for the purposes of the study, that the patient has not already been vaccinated against pneumococcal disease.

The study is divided into two phases:

- The "Observational" phase: Patients included during this period will receive their usual care.
- The "Interventional" phase: Patients included during this period will benefit, in addition to their usual care, from structured medico-pharmaceutical collaborative care. The aim of this collaborative approach is to promote the need for pneumococcal vaccination before patients are discharged from hospital.

The collaborative approach will take place prior to hospital discharge, as described

below:

- - The physician will provide the patient with a discharge prescription for vaccination.
  - A pharmacist will provide the patient with information about the pneumococcal vaccine, and explain the new treatments.

For all patients, during the 6 months following inclusion, the study investigator will ensure that the two pneumococcal vaccines (13 valent & 23 valent) have been dispensed correctly by your pharmacy, and that they have been administered correctly by the patient’s GP or home nurse (if applicable).

Once the patient has been discharged from hospital, neither you nor the patient will be contacted any more.

**RESEARCH-SPECIFIC PROCEDURES NOT INCLUDED IN STANDARD CARE**

The only intervention that is not part of usual care is structured medico-pharmaceutical collaborative care, which will be reserved exclusively for patients included in the interventional phase of the study.

**reSeArch Population**

The study population corresponds to adult patients admitted for full hospitalization in a surgical or medical ward, who have to undergo medication reconciliation, whose pneumococcal vaccination status is negative and whose risk of pneumococcal infection is high.

**CONSTRAINTS & OBLIGATIONS**

- the patient must be a member or beneficiary of a health insurance scheme.
- You certify that the patient is not participating in any ongoing Category 1 research, and that he/she has not participated in any Category 1 studies in the previous 3 months.
- You certify that the patient is not up-to-date with his/her pneumococcal vaccination.

**EXPECTED BENEFIT(S)**

For patients benefiting from collaborative medical-pharmaceutical management, the support offered as part of this study should make it possible to improve their anti-pneumococcal coverage and consequently avoid potentially serious infections.

In general, the medico-pharmaceutical collaboration will also help optimize drug management by improving detection of at-risk patients who have not been previously vaccinated. Better continuity of care can then be ensured in the patient pathway by promoting the smooth running of the outpatient vaccination process.

**FORESEEABLE RISK(S)**

There are no risks involved in participating in this study.

**COMPENSATION**

- No compensation will be paid for this study.
- Interventions specifically added to the usual care for the specific needs of the research will be fully covered. Participation in the research will not incur any additional costs over and above those you would incur for your usual care.

**WHAT ARE YOUR RIGHTS ?**

- The patient’s hospital pharmacist must provide all the necessary explanations concerning this research. If you wish to withdraw the patient from the study at any time, for whatever reason, he/she will continue to benefit from medical monitoring, and this will in no way affect his/her future surveillance.
- As part of the research, the patient’s personal data will be processed electronically to enable the results of the research to be analyzed regarding the objectives presented to you. The data controller is Nîmes University Hospital. The study doctor and other study personnel will collect information about the patient, including his/her health data, concerning his/her participation in the study. This information, known as "Personal Information", is recorded on forms provided by the sponsor. Only information that is strictly necessary for the treatment and purpose of the research will be collected, and this data will be kept for up to two years after the last publication of the research results or, if no publication has been made, until the final research report is signed. They will then be archived on paper or electronically for a period of 15 years from the end of the study, in accordance with current regulations. To ensure the confidentiality of the patient’s personal information, neither his/her name nor any other information that could directly identify him/her will be entered on the case report form or in any other file or sample that the study physician provides to the sponsor or the sponsor's authorized representatives. The patient will be identified only by a code and his/her initials. The code is used so that the study pharmacist can identify the patient if necessary.
- In accordance with the provisions of the French Data Protection Act (Act no. 78-17 of January 6, 1978 on Data Processing, Data Files and Individual Liberties, as amended by Act no. 2018-493 of June 20, 2018 on the protection of personal data) and the General Data Protection Regulation (EU Regulation 2016/679), you have the following rights with regard to the data we collect as part of this study:
- the right to request information on the processing of the patient’s data.
- the right to request rectification of the patient’s data if it is inaccurate or incomplete. While we examine your request, you have the right to restrict the processing of the patient’s data,
- the right to withdraw your consent or to object to the processing of the patient’s personal data, at any time, without having to justify your decision. No further data will be collected after you have withdrawn your consent.
- If you withdraw your consent or object to the processing of the patient’s data, you may request the deletion of the data already collected if there is no other legal requirement for their use. Please note, however, that the patient’s data that will have already been processed with your initial consent will be retained so as not to make it impossible or compromise the achievement of the research objectives (Articles 17.3.c & 17.3.d. of the General Data Protection Regulation).

You may exercise these rights by writing to the study's hospital pharmacist. The sponsor will respond to your requests as far as possible in accordance with its other legal and regulatory obligations and where required by law.

These rights may be exercised with the pharmacist who is following you as part of the research and who is aware of the patient’s identity.

You also have the right to object to the transmission of data covered by professional secrecy that may be used and processed as part of this research. You also have the right to access all the patient’s medical data, either directly or through the doctor or pharmacist of your choice, in accordance with article L1111-7 of the French Public Health Code. These rights may be exercised with the pharmacist who is following the patient as part of the research and who is aware of his/her identity.

The competent authorities and the sponsor or its authorized representatives may also require access to the patient’s medical records and study file, in order to verify the data collected in the course of the study.

The patient’s coded personal information may be used for other scientific research on pneumococcal vaccination or vaccination policy, always in compliance with applicable laws and regulations. In this context, research may be carried out by the promoter alone, or with one or more public or private institutions (including outside the European Union), or only by the latter. You may object to this re-use at any time by contacting the doctor who is treating the patient as part of this research, or the Data Protection Officer.

Should you have any further questions about the collection and use of the patient’s personal information, or about your rights in relation to this information, please contact the CHU de Nîmes Data Protection Officer (dpd@chu-nimes.fr) or the study pharmacist. The postal address of the DPO is as follows: DPD, CHU de Nîmes, place du Pr Debré 30029 Nîmes.

If, despite the measures put in place by the promoter, you feel that the patient’s rights have not been respected, you may file a complaint with the competent data protection supervisory authority in your country of residence (the CNIL for France) by writing to the following address: CNIL, TSA 80715, 3 Place de Fontenoy, 75334 Paris or directly on the CNIL website at [www.cnil.fr](http://www.cnil.fr).

In accordance with law n°2012-300 of March 5th, 2012 on research involving human persons:

- this research has obtained a favorable opinion from the Comité de Protection des Personnes EST IV and has been declared to the Agence Nationale de Sécurité du Médicament et des produits de santé (ANSM), an authorization from the CNIL will be requested and the NIR will be collected to query SNIRAM data.

- Nîmes University Hospital has taken out civil liability insurance with HDI GLOBAL SE (n° 0101242214029) (Tour Opus 12, La Défense 9, 77 Esplanade du Général de Gaulle - 92 914 Paris La Défense Cedex),

-people who have suffered prejudice as a result of participating in research can assert their rights before the regional commissions for conciliation and compensation of medical accidents,

- once the research is completed, you will be personally informed of the overall results by the patient’s pharmacist as soon as they are available, if you so wish.

- After reading this information note, please do not hesitate to ask the hospital pharmacist any questions you may have.

*Thank you for your attention.*

**Should you have any questions, please do not hesitate to ask the study coordinating pharmacist.**

| \| **Name and contact details of the coordinating investigator** \| \| --- \| \| Dr Florent DUBOIS  Pharmacy department  CHU de Nîmes  Tel : 04.66.68.31.04 \|   **Please be assured that the patient’s participation is extremely valuable to us. Thank you in advance for your support of medical research.** |
| --- | --- | --- |


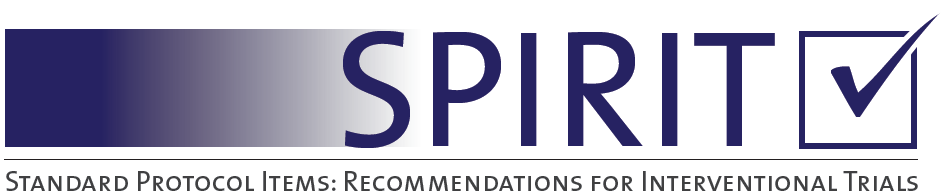


SPIRIT 2013 Checklist: Recommended items to address in a clinical trial protocol and related documents*

| Section/item | Item No | Description | Addressed on page number |
| --- | --- | --- | --- |
| **Administrative information** | | |  |
| Title | 1 | Descriptive title identifying the study design, population, interventions, and, if applicable, trial acronym | __1_____ |
| Trial registration | 2a | Trial identifier and registry name. If not yet registered, name of intended registry | ____4___ |
|  | 2b | All items from the World Health Organization Trial Registration Data Set | ____4____ |
| Protocol version | 3 | Date and version identifier | ____4____ |
| Funding | 4 | Sources and types of financial, material, and other support | __3_____ |
| Roles and responsibilities | 5a | Names, affiliations, and roles of protocol contributors | ___1; 3__ |
|  | 5b | Name and contact information for the trial sponsor | _____3__ |
|  | 5c | Role of study sponsor and funders, if any, in study design; collection, management, analysis, and interpretation of data; writing of the report; and the decision to submit the report for publication, including whether they will have ultimate authority over any of these activities | ___3____ |
|  | 5d | Composition, roles, and responsibilities of the coordinating centre, steering committee, endpoint adjudication committee, data management team, and other individuals or groups overseeing the trial, if applicable (see Item 21a for data monitoring committee) | ___NA___ |
| Introduction |  |  |  |
| Background and rationale | 6a | Description of research question and justification for undertaking the trial, including summary of relevant studies (published and unpublished) examining benefits and harms for each intervention | __5; 6___ |
|  | 6b | Explanation for choice of comparators | ___6; 7__ |
| Objectives | 7 | Specific objectives or hypotheses | ___6____ |
| Trial design | 8 | Description of trial design including type of trial (eg, parallel group, crossover, factorial, single group), allocation ratio, and framework (eg, superiority, equivalence, noninferiority, exploratory) | ____6__ |
| Methods: Participants, interventions, and outcomes | | |  |
| Study setting | 9 | Description of study settings (eg, community clinic, academic hospital) and list of countries where data will be collected. Reference to where list of study sites can be obtained | __7; 8___ |
| Eligibility criteria | 10 | Inclusion and exclusion criteria for participants. If applicable, eligibility criteria for study centres and individuals who will perform the interventions (eg, surgeons, psychotherapists) | __7; 8___ |
| Interventions | 11a | Interventions for each group with sufficient detail to allow replication, including how and when they will be administered | __9; 10__ |
|  | 11b | Criteria for discontinuing or modifying allocated interventions for a given trial participant (eg, drug dose change in response to harms, participant request, or improving/worsening disease) | __9; 10 __ |
|  | 11c | Strategies to improve adherence to intervention protocols, and any procedures for monitoring adherence (eg, drug tablet return, laboratory tests) | ___9; 10_ |
|  | 11d | Relevant concomitant care and interventions that are permitted or prohibited during the trial | ___NA___ |
| Outcomes | 12 | Primary, secondary, and other outcomes, including the specific measurement variable (eg, systolic blood pressure), analysis metric (eg, change from baseline, final value, time to event), method of aggregation (eg, median, proportion), and time point for each outcome. Explanation of the clinical relevance of chosen efficacy and harm outcomes is strongly recommended | ___8; 9__ |
| Participant timeline | 13 | Time schedule of enrolment, interventions (including any run-ins and washouts), assessments, and visits for participants. A schematic diagram is highly recommended (see Figure) | ____7___ |
| Sample size | 14 | Estimated number of participants needed to achieve study objectives and how it was determined, including clinical and statistical assumptions supporting any sample size calculations | ___11___ |
| Recruitment | 15 | Strategies for achieving adequate participant enrolment to reach target sample size | ____7___ |
| **Methods: Assignment of interventions (for controlled trials)** | | |  |
| Allocation: |  |  |  |
| Sequence generation | 16a | Method of generating the allocation sequence (eg, computer-generated random numbers), and list of any factors for stratification. To reduce predictability of a random sequence, details of any planned restriction (eg, blocking) should be provided in a separate document that is unavailable to those who enrol participants or assign interventions | ____6___ |
| Allocation concealment mechanism | 16b | Mechanism of implementing the allocation sequence (eg, central telephone; sequentially numbered, opaque, sealed envelopes), describing any steps to conceal the sequence until interventions are assigned | ______NA______ |
| Implementation | 16c | Who will generate the allocation sequence, who will enrol participants, and who will assign participants to interventions | ______NA______ |
| Blinding (masking) | 17a | Who will be blinded after assignment to interventions (eg, trial participants, care providers, outcome assessors, data analysts), and how | ___11___ |
|  | 17b | If blinded, circumstances under which unblinding is permissible, and procedure for revealing a participant’s allocated intervention during the trial | __NA____ |
| **Methods: Data collection, management, and analysis** | | |  |
| Data collection methods | 18a | Plans for assessment and collection of outcome, baseline, and other trial data, including any related processes to promote data quality (eg, duplicate measurements, training of assessors) and a description of study instruments (eg, questionnaires, laboratory tests) along with their reliability and validity, if known. Reference to where data collection forms can be found, if not in the protocol | ___11___ |
|  | 18b | Plans to promote participant retention and complete follow-up, including list of any outcome data to be collected for participants who discontinue or deviate from intervention protocols | ___NA_____ |
| Data management | 19 | Plans for data entry, coding, security, and storage, including any related processes to promote data quality (eg, double data entry; range checks for data values). Reference to where details of data management procedures can be found, if not in the protocol | ___9___ |
| Statistical methods | 20a | Statistical methods for analysing primary and secondary outcomes. Reference to where other details of the statistical analysis plan can be found, if not in the protocol | ___11___ |
|  | 20b | Methods for any additional analyses (eg, subgroup and adjusted analyses) | __11; 12_ |
|  | 20c | Definition of analysis population relating to protocol non-adherence (eg, as randomised analysis), and any statistical methods to handle missing data (eg, multiple imputation) | ______NA__ |
| **Methods: Monitoring** | | |  |
| Data monitoring | 21a | Composition of data monitoring committee (DMC); summary of its role and reporting structure; statement of whether it is independent from the sponsor and competing interests; and reference to where further details about its charter can be found, if not in the protocol. Alternatively, an explanation of why a DMC is not needed | ___11___ |
|  | 21b | Description of any interim analyses and stopping guidelines, including who will have access to these interim results and make the final decision to terminate the trial | ____NA____ |
| Harms | 22 | Plans for collecting, assessing, reporting, and managing solicited and spontaneously reported adverse events and other unintended effects of trial interventions or trial conduct | ___NA__ |
| Auditing | 23 | Frequency and procedures for auditing trial conduct, if any, and whether the process will be independent from investigators and the sponsor | __NA____ |
| Ethics and dissemination | | |  |
| Research ethics approval | 24 | Plans for seeking research ethics committee/institutional review board (REC/IRB) approval | ____2___ |
| Protocol amendments | 25 | Plans for communicating important protocol modifications (eg, changes to eligibility criteria, outcomes, analyses) to relevant parties (eg, investigators, REC/IRBs, trial participants, trial registries, journals, regulators) | __NA____ |
| Consent or assent | 26a | Who will obtain informed consent or assent from potential trial participants or authorised surrogates, and how (see Item 32) | Supplementary |
|  | 26b | Additional consent provisions for collection and use of participant data and biological specimens in ancillary studies, if applicable | ___NA__ |
| Confidentiality | 27 | How personal information about potential and enrolled participants will be collected, shared, and maintained in order to protect confidentiality before, during, and after the trial | ___11___ |
| Declaration of interests | 28 | Financial and other competing interests for principal investigators for the overall trial and each study site | ___2____ |
| Access to data | 29 | Statement of who will have access to the final trial dataset, and disclosure of contractual agreements that limit such access for investigators | ___11___ |
| Ancillary and post-trial care | 30 | Provisions, if any, for ancillary and post-trial care, and for compensation to those who suffer harm from trial participation | __NA___ |
| Dissemination policy | 31a | Plans for investigators and sponsor to communicate trial results to participants, healthcare professionals, the public, and other relevant groups (eg, via publication, reporting in results databases, or other data sharing arrangements), including any publication restrictions | __12___ |
|  | 31b | Authorship eligibility guidelines and any intended use of professional writers | __3____ |
|  | 31c | Plans, if any, for granting public access to the full protocol, participant-level dataset, and statistical code | ___NA__ |
| Appendices |  |  |  |
| Informed consent materials | 32 | Model consent form and other related documentation given to participants and authorised surrogates | Supplementary |
| Biological specimens | 33 | Plans for collection, laboratory evaluation, and storage of biological specimens for genetic or molecular analysis in the current trial and for future use in ancillary studies, if applicable | __NA____ |

*It is strongly recommended that this checklist be read in conjunction with the SPIRIT 2013 Explanation & Elaboration for important clarification on the items. Amendments to the protocol should be tracked and dated. The SPIRIT checklist is copyrighted by the SPIRIT Group under the Creative Commons “[Attribution-NonCommercial-NoDerivs 3.0 Unported](http://www.creativecommons.org/licenses/by-nc-nd/3.0/)” license.
